# Hearing Aid Amplification Schemes Adjusted to Tinnitus Pitch: A Randomized Controlled Trial

**DOI:** 10.3390/audiolres15060143

**Published:** 2025-10-22

**Authors:** Jose L. Santacruz, Emile de Kleine, Pim van Dijk

**Affiliations:** 1 Department of Otorhinolaryngology/Head and Neck Surgery, University Medical Center Groningen, University of Groningen, P.O. Box 30.001, 9700 RB Groningen, The Netherlands; joselopezsantacruz@gmail.com (J.L.S.); p.van.dijk@umcg.nl (P.v.D.); 2 Research School of Behavioral and Cognitive Neuroscience, Graduate School of Medical Sciences, University of Groningen, 9713 AV Groningen, The Netherlands

**Keywords:** tinnitus, hearing aids, notch therapy, tinnitus pitch, RCT, randomized control trial

## Abstract

**Background/Objectives**: Hearing aids can be used as a treatment for tinnitus. There are indications that this treatment is most effective when the tinnitus pitch falls in the frequency range of amplification of the hearing aid. Then, the hearing aid provides masking of the tinnitus. Alternatively, it has been suggested that a gap in the amplification around the tinnitus pitch would engage lateral inhibition and thereby reduce the tinnitus. **Methods**: To test these ideas, we conducted a randomized controlled trial. Patients were fitted with hearing aids using three different amplification schemes: (1) standard amplification according to the NAL-NL2 prescription procedure, (2) boosted amplification at the tinnitus frequency to enhance tinnitus masking, and (3) notch-filtered amplification at the tinnitus frequency to engage lateral inhibition and suppress tinnitus. The goal was to compare the boosted and notched amplification schemes to standard amplification. The primary outcome measure was tinnitus handicap as measured by the Tinnitus Functional Index (TFI). The trial was designed as a double-blind Latin square balanced crossover study. Eighteen tinnitus patients with moderate hearing loss were included. All of them were experienced hearing aid users. After two weeks of initial adaptation to the new hearing aids with standard settings, each setting was tried for four weeks. **Results**: There was an average reduction of 6.9 points on the TFI score after the adaptation phase, possibly due to a placebo effect. The TFI score did not differ significantly from the standard setting after using the notched or the boosted settings. Although notched amplification performed better than boosted amplification, this difference did not reach the clinical significance level. Regardless of the TFI outcomes, most participants had an individual preference for a particular setting. This preference was approximately uniformly distributed across the three amplification schemes. **Conclusions**: Notch-filtered and boosted amplification did not provide better tinnitus suppression than standard amplification. The individual preferences highlighted the importance of tailor-made approaches to hearing aid amplification in clinical practice. Further studies should explore the differences among patient’s tinnitus and their preference for a hearing aid setting.

## 1. Introduction

Tinnitus is described as ‘ringing’ or ‘hissing’ in the ears or the head in the absence of any external sound. About 15% of European adults experience tinnitus; however, as hearing status deteriorates with age, this proportion is larger in older adults [1]. The most common comorbidity of tinnitus is hearing loss, which has an odds ratio of 8.5 for developing tinnitus in the case of the Dutch population [2].

Neurophysiological studies suggest that tinnitus can be based on maladaptive adaptation to hearing loss [3,4,5]. Hence, some of the treatments aim to reverse the changes produced by such maladaptive adaptation. Hearing aids and other sound therapies for tinnitus are examples of this. These treatments may reverse the abnormal brain activity that could originate from acoustic deprivation by recalibrating central gain [6], preventing maladaptive neuroplasticity [7], or restoring connectivity in the auditory pathway [8,9].

A particular application of sound therapy is Tailor-Made Notched Music Training (TMNMT), which aims at reducing spontaneous activity in neurons by enhancing lateral inhibition from the frequencies above and below the tinnitus frequency [10,11]. These authors suggested that the initialization, development, and manifestation of tinnitus are due to a loss of lateral inhibition affecting the functioning of specific populations of auditory neurons. By creating a spectral gap at the frequency that matches the tinnitus pitch (referred to as the tinnitus frequency), lateral inhibition could be engaged and potentially ameliorate tinnitus. An application of this idea is the notched amplification in some hearing aids [12], which combines both the amplification of regular hearing aids and a notch filter centered at the user’s tinnitus frequency.

Hearing aids mainly increase the volume of external sounds, which improves the communication of users and, consequently, can help to reduce other tinnitus symptoms like stress or anxiety. But they also provide distraction from tinnitus and, more importantly, masking [13]. Masking might be the main cause for the reduction in tinnitus symptoms. The tinnitus literature suggests that the tinnitus frequency might have an influence on the result of sound-based therapies. Furthermore, recent evidence indicates that the intensity of the tinnitus percept itself can negatively impact speech comprehension, a key aspect of hearing aid benefit [14]. For example, previous studies suggested that the perceived pitch usually corresponds to frequencies where hearing is impaired [15,16,17]. It has been shown that masking is more likely to be achieved when the tinnitus pitch falls into the frequency range of the hearing aids [18,19,20]. In this context, it is reasonable to think of an opposite approach to the notch therapy discussed above: by boosting the amplification of the frequencies close to the tinnitus percept, masking might be enhanced and, as a consequence, bothersome tinnitus can be diminished.

The existing literature highlights the lack of high-quality evidence to support the clinical efficacy and effectiveness of hearing aids for tinnitus [21]. Shekhawat et al. [22] pointed out the scarce evidence for the effects of hearing aids in tinnitus management, underlining the low quality of the studies in the form of non-randomized controlled trials (non-RCTs). A similar conclusion was drawn in a recent scoping review on hearing aids and tinnitus [23] where, despite the positive results of most of the included studies, the authors emphasized the variability in the quality among these studies. Well-designed randomized controlled trials are necessary in tinnitus research to provide the highest grade of evidence quality for treatment efficacy, as is described in clinical guidelines [24].

Due to all the aforementioned reasons, the aim of this study was to conduct a randomized controlled trial (RCT) to test the effect on tinnitus of two amplification schemes in hearing aids by comparing them to a standard amplification scheme. The two test settings were notch amplification and boost amplification, both adjusted to the characteristics of each patient’s tinnitus frequency. A third setting, the standard amplification, served as a control. For this, 18 patients with tinnitus used hearing aids fitted with the three different programs during a total period of 14 weeks, switching the program with every 4 weeks of use in a randomized and double-blind procedure. Hearing aid fitting was ensured by real-ear measurements (REMs), and assessment of tinnitus and hearing aid benefit was carried out by means of questionnaires. Our aim was to specifically test the additional effect of a modified amplification strategy over standard amplification. Therefore, we tested these amplification strategies in participants who already use hearing aids.

## 2. Materials and Methods

### 2.1. Participants

The study was conducted at the ENT department of the University Medical Center Groningen (UMCG, 9700 RB, Groningen, The Netherlands). Of the 19 subjects who were enrolled, 18 completed the study. Two ways of recruiting participants were used. Most subjects were invited by an audiologist to participate after checking their medical record to ensure that they met the inclusion criteria. A few subjects joined after replying to the study advertisement on the UMCG website. Participants were included based on the presence of a moderate to moderate–severe degree of hearing loss, defined by the following characteristics: average pure tone audiometry (PTA) at 1, 2, and 4 kHz of at least 35 dB HL. This hearing criterion is the threshold used in the Netherlands for partial reimbursement of hearing aids. To prevent acoustic feedback in the hearing aids, all participants presented no more than 50 dB HL at 1 kHz. Additionally, in order to reduce variability due to differences in gain settings between both ears, participants were required to have symmetrical hearing loss, defined by no more than 15 dB HL of difference between both ears at each of the above frequencies. Moreover, all participants had chronic tinnitus (experiencing tinnitus for at least 6 months). The tinnitus of the participants had to be categorized as tonal so they could identify their tinnitus frequency during a pitch-matching task (see Section 4 of Materials and Methods). Furthermore, all participants were experienced hearing aid users, using their devices for at least 6 months before joining the study. All participants gave written informed consent before joining the study, which was approved by the ethics committee of the UMCG (METc 2021/128). In addition, this clinical trial was registered in the Dutch Trial Register, LTR, https://onderzoekmetmensen.nl/en (accessed on 1 September 2025), managed by the Central Committee of Research Involving Human Subjects (CCMO), for which the Ministry of Health, Welfare and Sport is responsible.

### 2.2. Questionnaires

After giving informed consent, participants received a series of questionnaires by mail that were sent back to the researchers with a return envelope. These questionnaires were the Tinnitus Functional Index [25], the Abbreviated Profile of Hearing Aid Benefit (APHAB [26]), and the Hyperacusis Questionnaire (HQ [27]). This initial round of questionnaires served to create a baseline. In order to monitor the participants’ progress during the trial, they had to complete these three questionnaires repeatedly throughout the entire study (see Section 2.5). The TFI was used to assess the effectiveness of the different treatments and their impact on the patients’ tinnitus. The purpose of the APHAB was to evaluate whether the different hearing aid settings had an impact on the patient’s hearing, independent of their tinnitus. Finally, the HQ was used to measure whether the settings had an impact on hyperacusis.

### 2.3. Hearing Aids and Amplification Schemes

Participants used the receiver-in-canal (RIC, size M) device Signia Pure 312 Nx 7 [28] with open click molds (vented) during the entire study. These hearing aids are specified (by the manufacturer) to be used in subjects with moderate-to-severe hearing loss. The devices had 20 frequency bands ranging from 0 to 12 kHz that could be adjusted individually through the fitting software Connexx v8.1.0 [28]. Participants could only increase or decrease the general gain of their devices if necessary, using their devices’ buttons or an app on their phones, but no changes in the frequency response were available to them. All the participants committed to not using their own devices until completion of the study. After enrollment, a document containing the instructions for using the devices was given to them.

Hearing aids were fitted according to three different amplification techniques:

- ***Standard amplification.*** In this setting, all available frequencies are amplified according to the standard clinical fitting using the formula NAL-NL2 [29]. This approach is consistent with the recommendation for patients with tinnitus and hearing loss in the ENT clinic of the UMCG.
- ***Notch amplification.*** This setting uses the previous fitting formula and, additionally, applies a notch filter centered at the participant’s tinnitus frequency. The rationale of this approach is based on lateral inhibition, by which excitation of neurons with a characteristic frequency close to the tinnitus frequency might suppress the tinnitus. The notch filter has a depth of 60 dB and a bandwidth of 0.5 octaves. The filter can be placed at each of 31 logarithmically distributed frequencies in the range of 0.250 to 8 kHz.
- ***Boosted amplification.*** The same fitting formula is applied. Additionally, the closest frequency band to the participant’s tinnitus frequency is amplified by 5 dB.

All three settings were stored in each participant’s profile in the software, ready to be loaded on the devices before each of the appointments.

### 2.4. Audiological Assessment and Hearing Aid Fitting

During the participant’s first visit to the clinic, pure tone audiometry from 0.250 to 8 kHz was performed. If they met the inclusion criteria, tinnitus pitch matching was carried out. For this, a multiple-choice method developed in a previous study was used [30], in combination with a MOTU UltraLite audio interface and a pair of Sennheiser HD660S headphones. This self-guided method allows the participant to choose between 22 available frequencies (ranging from 0.1 to 12 kHz), the one that matches their tinnitus pitch as closely as possible. The center frequency of this matching stimulus is referred to as the tinnitus frequency. Stimuli were presented in the contralateral ear in case of unilateral tinnitus and in the best hearing ear in case of bilateral tinnitus. The patient could choose between two main categories of stimuli: pure tones and noise. In the case of the latter, narrow-band noise with a bandwidth of ⅓ of an octave was used. Due to the limitation of 8 kHz as the maximum frequency at which the notch can be set in the study devices, participants with a higher tinnitus frequency were excluded. Inclusion of participants with noise-like tinnitus was possible if a dominant frequency could be determined by the pitch-matching test.

**Figure 1:**
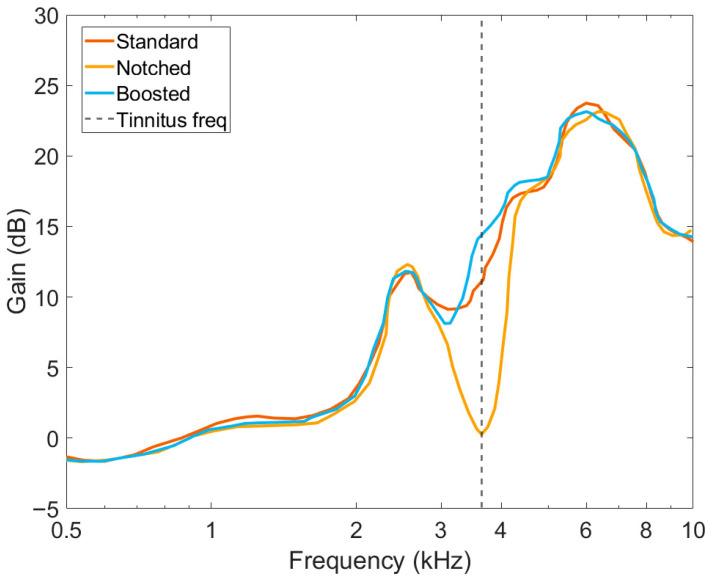
Real-ear measurement (REM) of the three settings of a participant with tinnitus at 3.5 kHz, which is shown as a dotted line. The curves represent the insertion gain responses of the different settings in the ear canal at 65 dB SPL using the ISTS stimulus.

With the obtained hearing thresholds and tinnitus frequency, a pair of hearing aids was fitted with the 3 settings according to Section 2.3. In order to ensure the correct fitting of the different settings, both objective and subjective measures are an important common practice in the clinic [13]. For this reason, some small adjustments of the NAL-NL2 formula were made until each participant was comfortable with the gain setting. All three settings were verified by means of real-ear measurements (REMs) using an Affinity 2.0 [31] and in-ear probes. After measuring the open ear response with white noise at 65 dB SPL, without removing the probes, hearing aids were placed in the participants’ ears. Then, the insertion gain of the three settings was measured by using the free-field International Speech Test Signal (ISTS [32]) at 55, 65, and 75 dB SPL. Figure 1 shows the insertion gain measure of the three settings from a participant whose tinnitus was matched at 3.5 kHz. The curves shown were obtained using 65 dB SPL as the loudness of the ISTS stimulus. To confirm that none of the settings affected the speech understanding of the participants, free-field speech audiometry at 60 and 70 dB SPL was measured. The real-ear measurements of the three settings were carried out during the first appointment for each participant to ensure the blinding of the study.

### 2.5. Study Design and Blinding

In order to maximize the power of the statistical tests considering the sample size, this study was designed as a crossover trial. An additional advantage of this design is the capacity to measure the response to a specific treatment in comparison to the same participant’s response to the other treatments [33]. In this sense, each participant becomes their own control subject, which is particularly interesting given the heterogeneity of treatment response in tinnitus. In long clinical trials such as this, washout periods can increase the dropout rates [34]. To avoid this and also potential carryover effects, characteristic of crossover studies, we opted for a *balanced Latin square* design [35]. Table 1 shows the treatment order of each group of three participants. The design contains all the possible combinations of treatment order in a balanced way. The sample size for this study was calculated following the indications for TFI repeated measures of Fackrell et al. [36], establishing a standard deviation of a difference of 7.2 points in the TFI, and α = β = 0.05.

This study was performed in a randomized controlled and double-blind manner. During the entire trial, none of the participants nor the researchers involved in the study had any knowledge of which setting was programmed in the hearing aids given to each participant at each appointment. A researcher not involved in this study designed a table with the participants’ identification number and their treatment orders, as in Table 1. This document was printed and kept in an envelope, unseen by the researchers. Prior to each participant’s appointment, 3 pairs of identical hearing aids were programmed according to their individual fitting, each of them with one of the settings (either standard, notched, or boosted). When a participant came to the clinic for either starting the trial or switching between programs, the envelope with the appointment order, the patient’s id, and the three pairs of devices in separate boxes were given to a person not involved in the study. The boxes were labeled to reflect the three settings. The hearing aids themselves were not labeled. The person, in a separate room, would take all the devices out of their boxes and only give the researcher the corresponding pair of devices to be handed to the participant. The other two pairs were reset. Thus, when giving the selected pair to the participant, neither the researcher nor the participant would be aware of the setting in the device. The study remained blinded until all participants completed the trial.

### 2.6. Procedure

**Figure 2:**
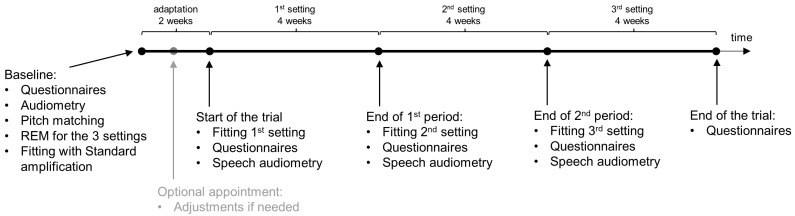
Study timeline. Each dot in the line represents an appointment. Performed tasks are listed underneath each appointment.

Participants were enrolled for 14 weeks, as shown in Figure 2. During this period, participants visited the clinic 5 or 6 times until completion of the study. The figure shows all the procedures that took place during each of the visits. In the first two weeks, the participants obtained the new devices with a regular NAL-NL2 fitting formula (standard amplification) for adaptation. If the subjects were not satisfied with the gain setting, they could come for an extra appointment prior to the start of the trial (optional appointment in Figure 2), where small adjustments of individual frequency bands could be made until the loudness was pleasant. After 2 weeks, the participants would start the randomized portion of the trial itself.

Once the trial started (after 2 weeks of adaptation), participants would visit the clinic once every 4 weeks to obtain a new pair of identical-looking devices, fitted with the corresponding setting. During each of these visits, the procedure was the same: filling in the three questionnaires, informal discussion on their hearing and tinnitus status, handing in the devices with the previous setting, obtaining the new ones, and undergoing speech audiometry. During the informal discussions, participants were asked whether they preferred the current setting compared to the previous one. Information about the usage time of the previous devices as logged by the devices would be stored, while ensuring the blinding. At the end of the trial, each participant ranked the settings in order of preference, ranging from worst to best in relation to their tinnitus status.

At the end of the trial, participants could keep the devices if they wished or go back to using their own hearing aids.

### 2.7. Statistical Analysis

Statistical analyses of the data were performed using both R (version 4.0.2) and SPSS (version 30.0). In order to avoid the bias of a potential placebo effect produced by joining a tinnitus study and using new hearing aids, the main analysis was carried out to measure the changes in the TFI score between the standard setting and the other two settings. Between-treatment comparisons were calculated using linear mixed-effects models. For this, all predictors and interactions between these were tested on an iterative process of model comparisons. Fixed effects were tested by using the expected mean square approach, which involves comparing the variance associated with the different predictors to the residual variance through the F-statistic and then determining their statistical significance. As to the random effects, we set up a model with a random intercept for the participant level. Conditional and marginal *R*^2^ were used to measure the proportion of explained variance [37]. Post hoc Tukey tests were used for multiple comparisons. The assumptions of linear mixed-effects models were verified (linearity, normality of residuals, homogeneity of variance, and no outliers).

In addition to the linear mixed-effects models with covariates, we conducted a supplementary Wilcoxon signed-rank test with Bonferroni correction to compare the raw TFI scores between treatments.

The main model was used for measuring tinnitus status and used the TFI score as the response variable. The following covariates (predictors) were tested for this model: hearing thresholds, speech audiograms (averaged free-field score at 60 and 70 dB SPL), tinnitus frequency (categorical variable of 22 possible values resulted from the pitch-matching method) and tinnitus type (tonal or noise-like), age, hyperacusis, average hours of use of the devices, and order of setting. Hearing thresholds were characterized as the average PTA for 2, 4, and 8 kHz. A similar analysis was conducted for both the HQ score and the APHAB score.

A post hoc analysis using linear mixed models was conducted to measure the impact on TFI when having the preferred setting as the main predictor, instead of each individual setting. The rest of the predictors tested remained the same as in the previous analysis. For this, the same procedure and variables were used.

## 3. Results

### 3.1. Demographics

Demographic variables and the results of the questionnaires are shown in Table 2. Participants were on average around 60 years old, and two-thirds of them were male. On average, the TFI scores at baseline indicated that tinnitus was a moderate problem for the participants [38]. For hyperacusis, six of the participants scored above 22 in the HQ questionnaire when starting the trial, which is considered to indicate hyperacusis [39]. The APHAB questionnaire scores showed an average benefit above 25, which confirmed the benefit of the hearing aid fitting [40]. One participant dropped out of the study during the adaptation period since they found the standard hearing aid setting (implementing NAL-NL2) unpleasant. A new participant joined the study as a replacement. Nine out of the eighteen participants made use of the optional appointment to adjust the gain settings during the adaptation period. No significant difference in the hours of use between the three settings was found.

### 3.2. TFI During the Adaptation Period

**Figure 3:**
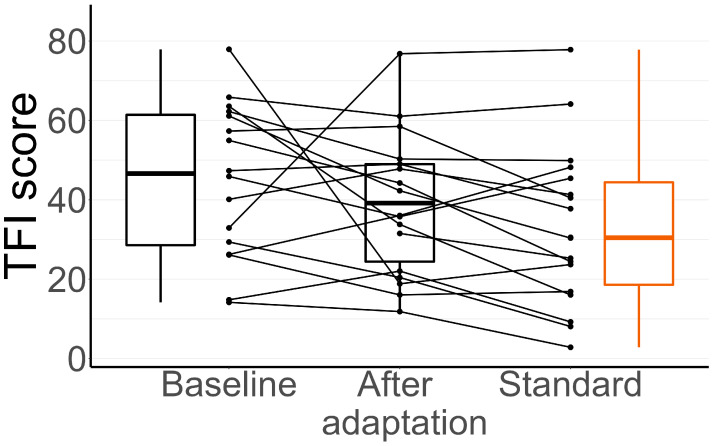
TFI scores of participants at baseline, adaptation stage, and after using the standard setting. After hearing aids were fitted at baseline, a two-week adaptation period started. Baseline and adaptation were sequential stages in the timeline of the trial, while standard setting occurred at different stages for each participant. Each participant is represented by dots that are connected. Median values are represented inside the boxplots.

The results of the TFI for the baseline, adaptation, and standard setting can be seen in detail in Figure 3. At all these time points, the standard setting was used. The figure shows a large variability in TFI scores and, in some cases, also large changes between stages. In addition, Table 3 shows the average reductions in the TFI scores between these stages of the trial. The differences in the TFI score during this period are summarized in Table 4, which shows the results of Welch’s *t*-test comparisons between baseline, adaptation, and the standard setting for the three questionnaires, with the corresponding Bonferroni corrections [41]. Note that the standard setting was not necessarily used right after the adaptation period for each participant, since the order of settings differs between participants. During the adaptation period of two weeks, every participant was using the standard setting. Although the mean score decreased, the *t*-test revealed no significant differences between baseline, adaptation, and the standard setting for the TFI results. The rest of the questionnaires also did not differ significantly between these periods. Two of the questionnaire forms at baseline went missing, which is the reason for the two missing data points at baseline in Figure 3.

### 3.3. TFI Results After Using Each Setting

**Figure 4:**
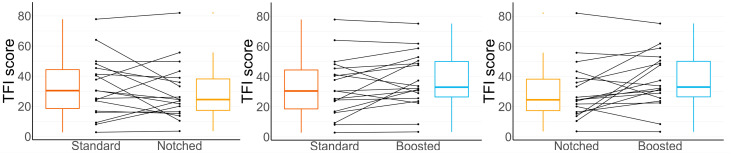
Line series graphs of the pair-wise TFI comparisons between the three settings. Each participant is represented by two dots connected with a line between the settings. Boxplots with median values are represented at both sides of each graph. Only the difference between notched and boosted (right panel) was significant.

To compare the outcomes after each setting, we used a linear mixed-effects model, as explained above. All the assumptions for linear mixed-effects models were met. Levene’s test of homogeneity of variance was not significant (*p* value = 0.495). Residuals were normally distributed and no outliers were observed. Figure 4 shows the pairwise comparisons between TFI scores of the three settings. As shown in the figure, TFI score changes varied across participants, with some patients exhibiting differences of nearly 30 points between settings.

The results of the fixed effects of the mixed model for the TFI score are shown in Table 5. This model included the standard as a reference and considered the effects of all the predictors mentioned in Section 2.7. No significant differences in the change in the TFI were found for the notched or boosted settings; therefore, the test failed in the rejection of the null hypothesis. While tinnitus frequency contributed significantly to the changes in the TFI, this was not the case for the type of tinnitus (tonal or noise-like) or the interaction between tinnitus frequency and the type of tinnitus. The tinnitus frequency contributed negatively to the TFI increase, meaning that participants with lower tinnitus pitch achieved poorer results. The HQ score also had a significant effect on the result of the TFI. In this case, participants with lower HQ scores performed better than those with higher HQ scores. The results of the model fit indicated that both the fixed predictors and individual differences across participants contributed substantially to the explained variance. For multiple comparisons, post hoc Tukey contrasts on the settings were checked, and the results are shown in Table 6. There was no statistically significant difference in TFI reduction between the notched or boost settings and the standard setting. A significant difference between notched and boosted settings was found, with the notched setting giving a higher TFI reduction. This difference did not reach the clinical significance level of 14 points for the TFI [35]. Regardless of amplification strategy, hearing aid benefit as measured by the APHAB was comparable across strategies.

The results of the Wilcoxon signed-rank test are shown in Table 7. This non-parametric test, which compared the differences in the TFI between the three settings without taking into account any covariates, resulted in non-significant differences between the settings.

### 3.4. Preferred Setting

**Figure 5:**
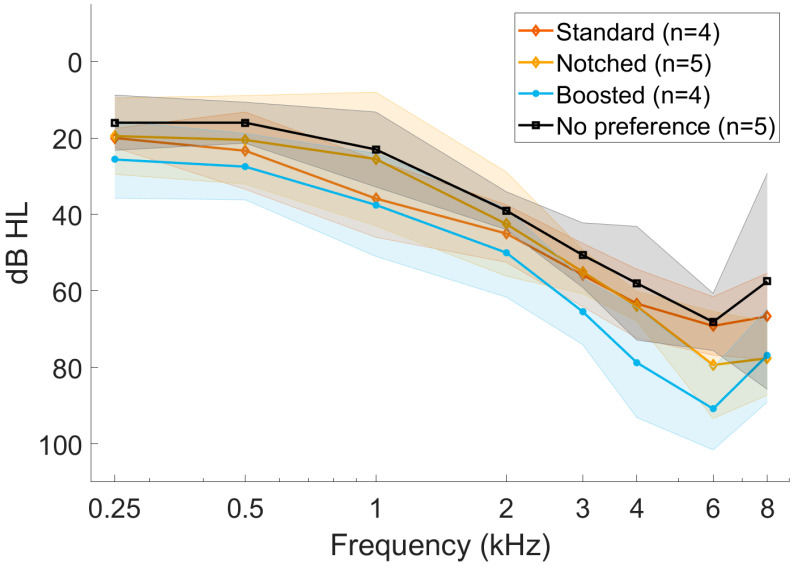
Hearing thresholds of participants when arranging them by preferred setting. Each group size is expressed by *n*. Results are represented as mean values ± standard deviations as shaded contours.

There was no consistent preference for a setting at the end of the trial. Thirteen participants expressed a preference for one of the three settings, whereas five participants did not have a preference. Figure 5 shows the hearing thresholds of the participants when these are grouped by preferred setting. Although some differences in thresholds in the high-frequency range can be seen, no significant difference in PTAs between the four groups was found.

**Figure 6:**
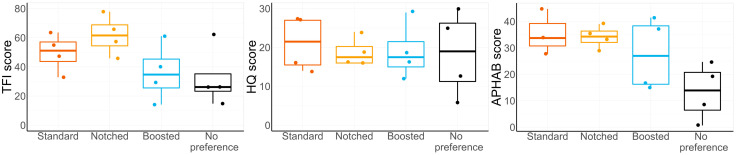
Results of the questionnaires at baseline when arranging the participants by preferred setting.

Figure 6 shows the results of the questionnaires at baseline, arranged by preferred setting at the end of the trial. The participants who had no preferred setting scored lower on average in the APHAB questionnaire at the beginning of the study (a lower score meaning that they experienced less benefit from their hearing aids). Participants who preferred the standard or notched settings had a higher TFI score when starting the trial. Given the low number of subjects in each group, no further statistical analysis was conducted.

**Figure 7:**
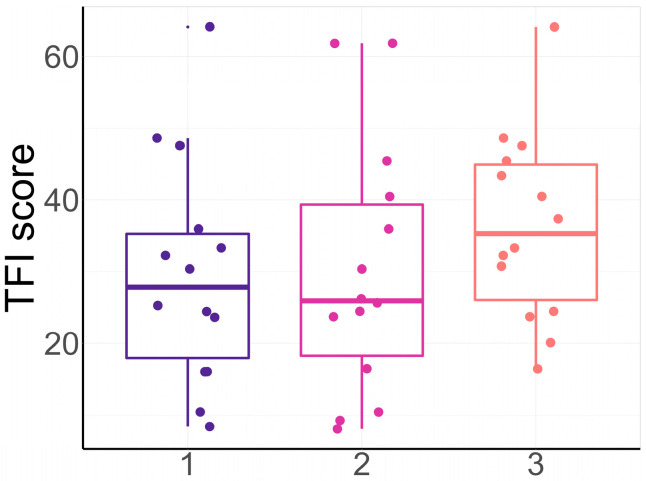
TFI scores of all subjects after a certain setting, ordered by preferred setting. The numbers on the x axis refer to the order of preference, ranging from 1 (most preferred setting) to 3 (least preferred setting).

A Chi-squared test between the preferred settings and the settings that obtained the lowest TFI score for each participant resulted in a Chi-square statistic of 11.0 (*p*-value = 0.09). Nine out of the eighteen participants preferred the setting that also reduced their TFI score the most. Figure 7 shows the TFI scores for the different preferences in order of the participants who did have a preference, with 1 being the most preferred setting and 3 being the least preferred setting. A pairwise comparison resulted in no significant difference in the TFI score between preferences.

## 4. Discussion

In this study, we investigated the efficacy of two amplification strategies in tinnitus treatment and compared them to a regular amplification scheme. The settings of the tested strategies were adjusted to each patient’s tinnitus frequency. The amplification schemes were fitted using the standard NAL-NL2 formula as a reference. For the control setting (standard amplification), no modifications were made. For the notched setting, a notch filter was applied at the center of the participant’s tinnitus frequency. For the boosted setting, a boost of 5 dB was applied at the tinnitus frequency. Overall, the TFI score was reduced by 6.9 points on average after 2 weeks of adaptation, and although not significant, it remained reduced after using any of the three settings. The comparison between settings revealed no significant reduction in the TFI score in comparison to the standard setting. Despite the TFI score after notched amplification being lower than after boost amplification, this difference did not reach the required clinical significance for the TFI. The HQ and APHAB questionnaires did not reveal any significant changes in hyperacusis or hearing aid benefit after using any of the tested settings. Notably, the hearing aid benefit was comparable across all amplification settings. The primary objective of this study was to determine whether the notch or boost amplification provided superior outcomes compared to the standard amplification, which was not supported by the results.

It is important to note the discrepancy between the results of the linear mixed-effects model and the simpler nonparametric test. While the linear mixed-effects model, which included covariates, resulted in a significant difference between two of the settings, the unadjusted nonparametric test did not reveal any significant difference. This contrast highlights the influence that the covariates have in the analysis, such as the tinnitus frequency and the HQ score, which suggests that these factors could influence the treatment outcomes.

The initial improvement in TFI scores during the initial 2-week adaptation period (Figure 3) may be due to a placebo effect. The placebo effect has been explored in the tinnitus literature [21] and, more generally, in the context of hearing aid studies [42]. Dawes et al. [42] highlighted the importance of double-blind studies to reduce the placebo effect in hearing aid studies, which this study succeeded in. Nevertheless, we cannot exclude the possibility that the effects in the adaptation period were due to the higher quality of the devices compared to the previous ones, which might have affected the way the participants perceived their tinnitus during the adaptation process.

Sound-based therapies for tinnitus have shown some promising results. These therapies apply either a sound that includes the tinnitus frequency [19,20] or they place a notch filter at the tinnitus frequency, thus presenting sounds that surround the tinnitus in the frequency domain [10,11]. In the first approach, masking of the tinnitus is the potential mechanism of tinnitus suppression. This approach is similar to the standard and boosted amplification schemes in the current hearing aid trial. In the second approach, lateral inhibition is the proposed suppression mechanism, where sounds with frequencies surrounding the tinnitus frequency inhibit neural activity associated with the tinnitus. When hearing aids are used as sound therapy, the sounds presented to the ear are an amplified version of the sound in the environment. Its effect on tinnitus is rather mixed. Some patients achieve masking of their tinnitus [19] but others only obtain it minimally [12]. The result of Marcrum et al. [12] was similar to ours, in that the differences between standard and notch amplification were non-significant. Since clinically significant levels were not reached for this comparison [36], future studies should not disregard the boost amplification as a treatment for tinnitus, considering that a certain portion of patients (22%) in our study did prefer this amplification strategy over the others (Figure 6).

The importance of patient preferences in hearing aid use has been previously highlighted by another randomized controlled trial. Humes et al. [43] found that participants who selected over-the-counter hearing aid interventions (limited to three common fitting formulas) achieved outcomes comparable to those of participants fitted according to best-practice audiological care, which included orientation, counseling, and real-ear measurements. For clinical application of the various hearing aid settings, it is important to identify which features determine the preference for one setting over another in individual patients. In our study, preference for one setting or no preference at all was equally distributed across the participants. The speech audiometry performed at baseline did not reveal any differences in speech understanding between the three settings; therefore, we do not consider speech understanding as a potential factor in the decision making. Despite the setting preferences being equally distributed and the fact that the groups based on preferred settings were too small to come to a conclusion, the between-groups differences in questionnaire scores at baseline suggest that a following study could further explore the correlation between setting preference and questionnaire scores before treatment in-depth.

During informal discussions at the appointments, patients would describe their reasons to opt for one specific setting over another one, mainly influenced by how their tinnitus percept evolved during the last few weeks but also by their stress or anxiety levels during the same period, which are well-known correlated factors of tinnitus [44]. Differences in daily life routines may have had an impact on the decision making. For instance, participants with active lifestyles, with more exposure to noisy environments such as public places, or simply by listening to the radio or the tv at home might obtain a bigger benefit when using any of the settings. In contrast, participants whose lifestyles are more quiet and whose activities require less listening effort might not be able to distinguish between settings. Some participants used the devices in a more augmented way, by listening to podcasts or audiobooks through the Bluetooth connection, which shaped the sound according to the gain settings. Ultimately, hearing aids, and especially those with tinnitus settings, need to be exposed to the circumstances that are relevant in the life of a patient in order to determine a preference for a specific setting. Altogether, a discussion of systemic, non-auditory factors is warranted, as these vary among tinnitus patients and may influence their experiences and preferences. This reinforces the concept that each patient’s unique tinnitus profile, shaped by both central and systemic factors [45], ultimately guides their preference for a particular hearing aid setting.

Some limitations must be acknowledged. Tinnitus subjectiveness imposes the use of questionnaires for assessing tinnitus relief. The TFI in particular measures the impact of tinnitus during the week prior to the appointment, which leaves a longer period of time without evaluation in between appointments. This might present a bias in the assessment, as was pointed out by some participants during the appointments. Sometimes, participants would describe a couple of bad initial weeks after a new fitting and substantial tinnitus relief during the third or fourth week, right before the assessment. This study covered the comparison between two different settings and a control setting over a total period of 14 weeks. The number of comparisons might have had an effect on the significance of the results. Future studies on specific hearing aid strategies might benefit from simplifying the study design by prolonging or maintaining the duration of it and reducing the number of tested settings to one.

On a different note, each setting was tested for a period of one month. Studies involving hearing aids typically aim for a period of at least three months to achieve significant improvements in tinnitus relief, although in most studies, naive hearing aid users are studied [46]. Lastly, the two tested settings in this study (notched and boosted amplification) required a tinnitus pitch-matching procedure for the correct fitting. Despite using a reliable pitch-matching method, it is still possible that the notch filter and the boost filter were applied in the wrong frequencies for some participants. This is a feasible scenario due to the subjectivity and complexity of a pitch-matching test. Additionally, it is worth mentioning that the 8 kHz limitation of the notch setting might also play a role in the results we observed, as it remains unclear how patients with tinnitus at higher frequencies might respond.

Nevertheless, some strengths of this study can also be acknowledged. Most of the studies on hearing aids in tinnitus relief are conducted with participants who never used hearing aids before. In our study, we included experienced hearing aid users (with at least 6 months of use) in order to diminish the initial improvement characteristic of receiving a new treatment. This approach allowed us to assess whether individuals who had already experienced tinnitus-related benefit from their hearing aids could experience additional improvement with notch or boost amplification strategies. The study design (a balanced and double-blind crossover study) allowed for minimizing the risk of confounding, increasing the power of statistical tests performed with a relatively low sample size [47]. We consider that these design decisions strengthened our results, and we encourage researchers to design tinnitus studies in a similar way. Future trials with hearing devices might benefit from the blinding procedure laid out in this study.

The tinnitus literature often states the necessity of RCTs in the context of tinnitus treatment [24]. There is a consensus in all the European guidelines with regard to the use of hearing aids in tinnitus treatment when patients suffer from hearing loss. Positive results are present in most studies; however, the quality of the evidence on the effectiveness of hearing aids for tinnitus relief is currently insufficient [23]. The current study was designed to shed more light on the efficacy of hearing aids for tinnitus treatment and, in particular, to assess the effectiveness of devices adapted to the patient’s tinnitus frequency. Our study showed that a notched or boosted amplification setting did not significantly improve the patient’s tinnitus compared to a standard setting. Individual preferences were different between participants, showing that a tailor-made approach to hearing aid amplification for tinnitus treatment is important in clinical practice. Further studies should explore the differences among patients’ tinnitus and their preferences for hearing aid settings. Moreover, hearing aid trials might benefit from the blinding procedure used in this study.

## Figures and Tables

**Table 1:** Balanced Latin square design, crossover study. Each row represents a group of 3 participants. Each group used the settings in the stated order.

Subjects	Treatment Order		
3	Standard	Notched	Boosted
3	Notched	Boosted	Standard
3	Boosted	Standard	Notched
3	Standard	Boosted	Notched
3	Notched	Standard	Boosted
3	Boosted	Notched	Standard

**Table 2:** Demographic characteristics and results of the questionnaires at the beginning of the trial. Each questionnaire is accompanied by its own subscales. Mean values and standard deviation are presented. * the APHAB score reflects the benefit of the hearing aids that participants used before entering the trial.

Demographic Data			
Number of subjects (n)			18
	Sex—n		
		male	12
		female	6
Age (years)			60.7 ± 12.7
Speech audiometry with hearing aids (% correct)			
	at 60 dB SPL		87.7 ± 7.9
	at 70 dB SPL		95.5 ± 5.5
PTA at 2, 4, and 8 kHz (dB HL)			57.3 ± 9.0
Questionnaires			
	**TFI score**		46.1 ± 13.2
		Intrusiveness	44.9 ± 19.4
		Sense of control	52.7 ± 24.5
		Cognition	38.3 ± 25.3
		Sleep	40.4 ± 31.4
		Auditory	56.2 ± 27.2
		Relaxation	58.3 ± 28.3
		Quality of life	40.7 ± 21.5
		Emotional distress	28.3 ± 22.3
	**HQ score**		19.3 ± 6.9
	APHAB score *		27.1 ± 12.7
		Ease of communication	30.3 ± 16.1
		Reverberation	23.3 ± 14.6
		Background noise	27.1 ± 14.7
		Aversiveness	−11.9 ± 16.6
Tinnitus			
	Frequency (kHz)		5.1 ± 3.0
	Type—n (pure tone/noise-like)		6/12
Hours of use per day			
	Standard setting		10.5 ± 4.5
	Notched setting		8.5 ± 5.5
	Boosted setting		9.8 ± 4.9

**Table 3:** Mean reductions (and standard deviations) of the TFI scores between stages of baseline and adaptation. Standard setting is included as a reference for the settings tested.

Comparison	Mean Reduction in the TFI Score (Points)
Baseline—Adaptation	6.9 ± 2.0
Baseline—Standard	12.0 ± 0.4
Adaptation—Standard	5.1 ± 2.4

**Table 4:** Welch’s *t*-test comparisons of the questionnaire’s scores at baseline, during the adaptation period, and after using the standard setting. Adjusted *p*-values for Bonferroni correction are shown.

Parameter	Comparison	df	*p* Value	Adjusted *p* Value
TFI score	Baseline—Adaptation	30.4	0.28	0.84
	Baseline—Standard	31.6	0.08	0.24
	Adaptation—Standard	33.4	0.41	1
HQ score	Baseline—Adaptation	31.5	0.89	1
	Baseline—Standard	32	0.83	1
	Adaptation—Standard	33.7	0.92	1
APHAB score	Baseline—Adaptation	44.6	0.52	1
	Baseline—Standard	25.40	0.31	1
	Adaptation—Standard	26.5	0.11	1

**Table 5:** Results from a mixed-effects analysis of the effect of various predictors on the TFI. Only fixed effects of the predictors are shown. numDF = number of degrees of freedom; Estimate = mean change in the TFI score; Std. error = standard error of the TFI score change; *p* value = significance level. Goodness-of-fit is represented by R^2^ conditional = proportion of variance in the TFI score that is explained by both the fixed and random effects; R^2^ marginal = proportion of variance in the TFI score that is explained by only the fixed effects.

Predictor	numDF	Estimate	Std. Error	*p* Value
setting—notched	2	−3.2	9.8	0.28
setting—boosted	2	3.7	3.0	0.21
tinnitus type—noise-like	1	0.4	8.0	0.92
tinnitus frequency	1	−21.0	9.9	<0.05
tinnitus frequency × tinnitus type	1	20.8	10.5	0.06
HQ score	1	1.2	0.3	<0.001
			R^2^ conditional	R^2^ marginal
Model performance			0.78	0.39

**Table 6:** Post hoc Tukey contrasts of the settings for the TFI model. Estimate = mean change in the TFI score; Std. error = standard error of the TFI score change; *p* value = significance level.

Comparison	Estimate	Std. Error	Adjusted *p* Value
Notched—Standard	−3.2	3.0	0.516
Boosted—Standard	3.7	3.0	0.414
Boosted—Notched	7.0	3.0	0.047

**Table 7:** Results of the Wilcoxon signed-rank test of the TFI scores after using each setting, using Bonferroni correction for multiple comparisons. *p* value = significance level.

Comparison	Adjusted *p* Value
Notched—Standard	1.0
Boosted—Standard	0.34
Boosted—Notched	0.25

## Data Availability

The data presented in this study are available on request from the corresponding author due to privacy and legal reasons.

## Abbreviations

The following abbreviations are used in this manuscript:

TMNMT	Tailor-Made Notched Music Training
UMCG	University Medical Center Groningen
CCMO	Central Committee of Research Involving Human Subjects
REM	Real-Ear Measurement
RCT	Randomized Controlled Trial
PTA	Pure Tone Audiometry
RIC	Receiver In Canal
TFI	Tinnitus Functional
APHAB	Abbreviated Profile of Hearing Aid Benefit
HQ	Hyperacusis Questionnaire
ISTS	International Speech Test Signal
